# Metastatic Hepatocellular Carcinoma Presenting as a Large Abdominal Mass and Secondary Varicocele in a Hepatitis B Patient: A Case Report

**DOI:** 10.7759/cureus.85933

**Published:** 2025-06-13

**Authors:** Mmahaletchumy Manoharan, Muhammad Hamza Shahid, Daniyal Abbasi, Lohana Pooja, Abdul Haseeb Hasan

**Affiliations:** 1 General Surgery, University of North Sumatra, Medan, IDN; 2 Internal Medicine, Akhtar Saeed Medical and Dental College, Lahore, PAK; 3 Internal Medicine, Akbar Niazi Teaching Hospital, Islamabad, PAK; 4 Internal Medicine, Liaquat University of Medical and Health Sciences, Jamshoro, PAK; 5 Internal Medicine, Mayo Hospital, Lahore, PAK

**Keywords:** abdominal mass, atypical presentation, case report, exophytic liver tumor, gastrointestinal bleeding, hepatitis b, hepatocellular carcinoma, varicocele

## Abstract

We report a rare and aggressive presentation of hepatocellular carcinoma in a 50-year-old man with a brief history of hepatitis B infection. The patient presented with a rapidly enlarging epigastric mass, recurrent hematemesis, and a newly developed left-sided varicocele. Imaging revealed a large exophytic hepatic mass with evidence of vascular displacement and intrahepatic metastases. Histopathological examination confirmed a diagnosis of poorly differentiated hepatocellular carcinoma. A multidisciplinary tumor board recommended palliative surgical debulking followed by systemic therapy to address tumor burden and improve quality of life. This case highlights the diagnostic and management challenges associated with atypical presentations of hepatocellular carcinoma in patients with minimal liver disease history.

## Introduction

Hepatocellular carcinoma (HCC) is the most common primary malignancy of the liver, accounting for over 80% of liver cancer cases globally [1]. It often arises in the setting of chronic liver disease, particularly cirrhosis due to hepatitis B virus (HBV), hepatitis C virus (HCV), or non-alcoholic fatty liver disease. HCC is typically diagnosed in the sixth or seventh decade of life and frequently presents with nonspecific symptoms or is detected during routine surveillance in high-risk populations [2]. While intrahepatic tumors are the norm, large exophytic masses and extrahepatic manifestations are rare. Atypical presentations may lead to diagnostic delays, especially in non-cirrhotic patients or those with subtle clinical signs. Recognizing such variants is crucial to initiate timely investigations and management.

This case aims to highlight a rare and aggressive presentation of HCC in a 50-year-old man with a relatively short history of hepatitis B infection. It focuses on the diagnostic challenges posed by an exophytic liver mass mimicking an abdominal tumor and a secondary varicocele due to venous compression. By sharing this case, we aim to raise clinical awareness about atypical HCC presentations and emphasize the importance of early imaging and multidisciplinary evaluation in patients with known risk factors.

## Case presentation

A 50-year-old South Asian man, previously healthy and a known HBV carrier for the past two years, presented to our surgical outpatient department with complaints of progressive abdominal distension over one month and multiple episodes of hematemesis in the preceding three days. He reported a sudden onset of an epigastric mass that had progressively enlarged, resulting in discomfort, early satiety, and intermittent dull abdominal pain. The vomiting of fresh blood, occurring 3-4 times daily, was accompanied by mild chest tightness and one episode of melena. The patient also reported a weight loss of approximately 10-15 kg within a month, profound fatigue, anorexia, and reduced physical capacity. There was no history of fever, jaundice, rash, or generalized body aches.

The patient had no known comorbidities, history of alcohol consumption, or prior hospitalizations. Notably, his mother had died of HCC 15 years ago. Given the patient's HBV status and strong family history, HCC was suspected. On general examination, the patient appeared pale and fatigued but was hemodynamically stable. Abdominal examination revealed a protuberant mass in the epigastrium, oval in shape, tender on deep palpation, and mobile in the transverse plane. The overlying skin was pinchable, and the mass decreased in prominence when the abdominal muscles were contracted, indicating it was not adherent to the muscle or skin. No ascites or stigmata of chronic liver disease were observed.

Genital examination revealed a swelling of the left testicle resembling a "bag of worms". The transillumination test was negative, and no scrotal tenderness or overlying skin changes were noted. No palpable abdominal or inguinal lymphadenopathy was appreciated initially, although later imaging identified several subcentimetric nodes. Laboratory investigations in Table 1 revealed severe anemia with a hemoglobin level of 3.5 g/dL and elevated urea, suggestive of upper gastrointestinal bleeding. Liver function tests showed raised aspartate aminotransferase (AST) (56.5 U/L) and alkaline phosphatase (ALP) (194 U/L), while alanine aminotransferase (ALT) remained within the normal range. Alpha-fetoprotein (AFP) levels were markedly elevated at 50,090 ng/mL.

**Table 1:** The baseline laboratory investigations in a patient with metastatic hepatocellular carcinoma WBC: white blood cell; AST: aspartate aminotransferase; ALT: alanine aminotransferase; ALP: alkaline phosphatase; AFP: alpha-fetoprotein; INR: international normalized ratio

Parameter	Result	Reference range
Hemoglobin	3.52 g/dL	13-18 g/dL
WBC count	11.3×10⁹/L	4-11×10⁹/L
Platelets	457×10⁹/L	150-400×10⁹/L
AST	56.5 U/L	≤40 U/L
ALT	14.5 U/L	≤40 U/L
ALP	194 U/L	89-120 U/L
Urea	118 mg/dL	10-50 mg/dL
Serum creatinine	0.92 mg/dL	0.5-0.9 mg/dL
AFP	50,090 ng/mL	<20 ng/mL
INR	1.06	0.9-1.3

Ultrasound revealed a non-encapsulated, heterogeneously hypoechoic mass measuring approximately 13.3×15.6×11.9 cm arising from the left lobe of the liver, exhibiting a central hypoechoic scar and mixed vascularity. Liver margins were smooth, with no evidence of intrahepatic or extrahepatic biliary dilatation. The rest of the liver parenchyma appeared unremarkable.

**Figure 1:**
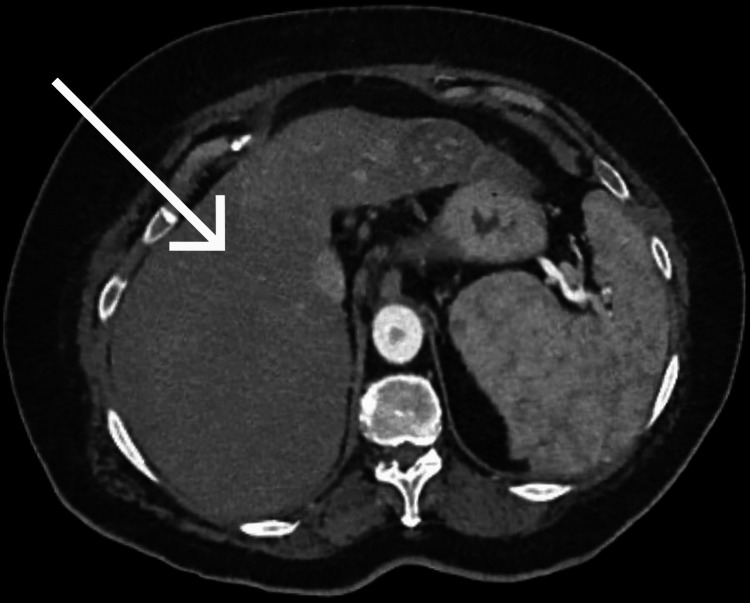
Triphasic CT scan showing a large exophytic heterogeneously enhancing mass centered in the midepigastric region, as marked by the arrow

Triphasic CT scan, as given in Figure 1, showed a large exophytic heterogeneously enhancing mass centered in the midepigastric region measuring 13×18.9×16 cm. The lesion exhibited arterial enhancement (54 HU), mild portovenous enhancement (45 HU), and washout on delayed images, consistent with classic radiologic features of HCC. The mass was inseparable from the anterior wall of the stomach and displaced the celiac axis and superior mesenteric artery. It abutted the left lobe of the liver, with preserved fat planes, and had indistinct fat planes anteriorly with the abdominal wall. Additional hypodense lesions were noted in liver segments IVb (2×3 mm) and VII (11×7.5 mm), raising suspicion for intrahepatic metastasis. Multiple subcentimetric mesenteric and paraaortic lymph nodes were also observed. No ascites was present.

**Figure 2:**
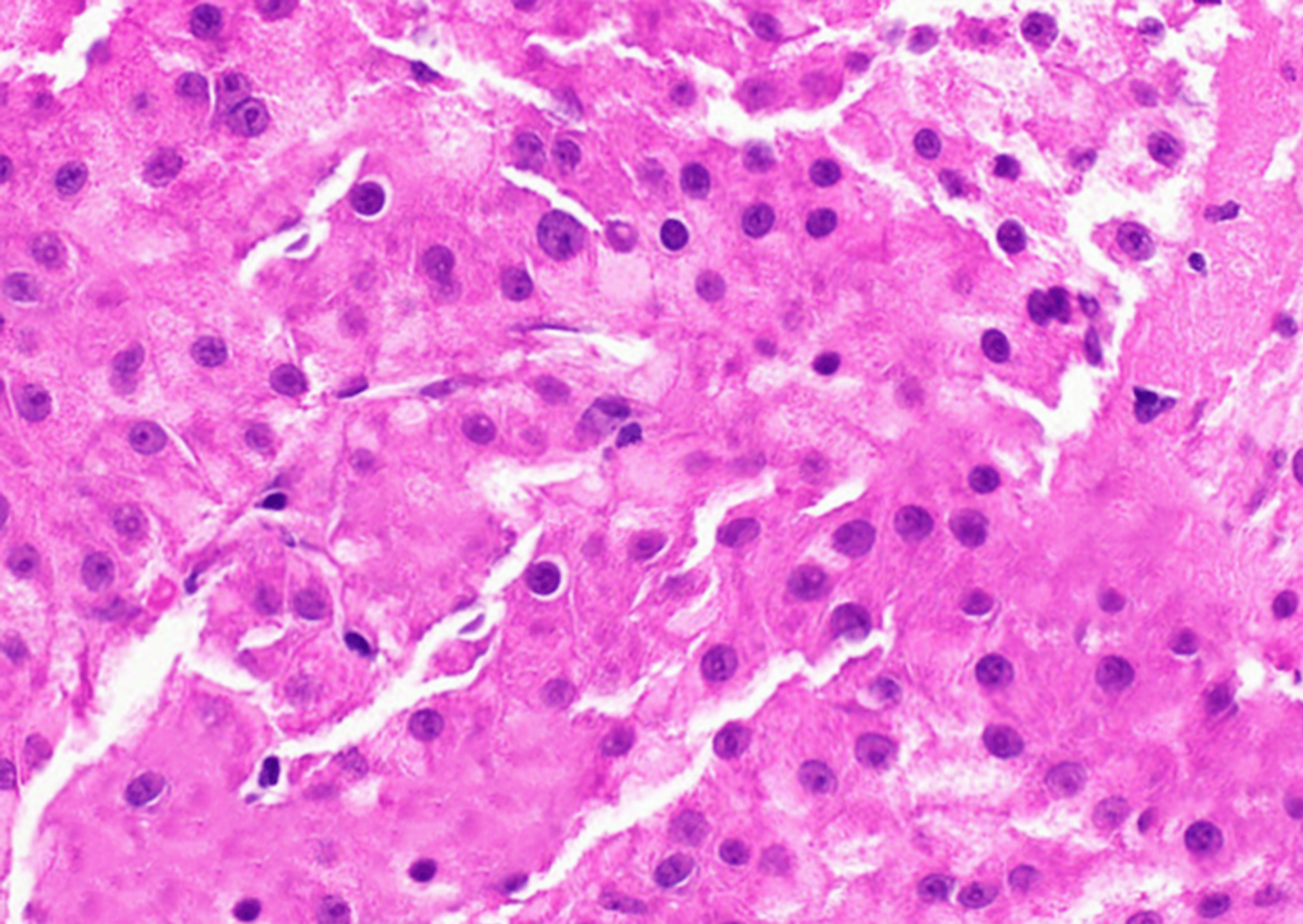
Sheets of pleomorphic, hyperchromatic malignant hepatocytes with prominent nucleoli, eosinophilic to clear cytoplasm, and frequent mitoses

Scrotal Doppler revealed a grade IV left-sided varicocele with venous reflux, likely secondary to extrinsic compression of the left testicular vein by the large abdominal mass. Esophagogastroduodenoscopy (EGD) demonstrated multiple esophageal varices with active bleeding. Endoscopic variceal band ligation was performed successfully, with no post-procedure complications. CT-guided biopsy of the liver mass was performed. Histopathological examination of the core tissue revealed sheets and nests of highly pleomorphic, hyperchromatic malignant hepatocytes with conspicuous nucleoli, abundant eosinophilic to clear cytoplasm, and frequent mitotic figures. The features are shown in Figure 2. Intervening blood vessels were noted. These findings confirmed the diagnosis of poorly differentiated HCC.

Based on clinical presentation, imaging, serological markers, and histopathology, a final diagnosis of advanced-stage, metastatic HCC arising from the left hepatic lobe was established. Lymph node involvement and metastatic lesions in liver segments IVb and VII further supported the advanced nature of the disease.

A multidisciplinary tumor board, comprising a general surgeon, a hepatobiliary specialist, an oncologist, a urologist, and a palliative care team, was convened to discuss the management plan. Owing to the tumor's size, vascular proximity, and early metastases, curative resection was not feasible. However, palliative surgical debulking was proposed to relieve mass effect symptoms and improve quality of life, followed by systemic therapy with sorafenib or lenvatinib, depending on tolerability and response. The patient and his family were counseled extensively regarding the disease progression, prognosis, and management options. He consented to proceed with palliative surgery, with preoperative optimization currently underway.

## Discussion

HCC is the most common primary malignancy of the liver [3]. It typically arises in the setting of cirrhosis or chronic hepatitis B or C infections, with most patients presenting with vague symptoms such as right upper quadrant discomfort, fatigue, or features of decompensated liver disease [4]. In contrast, our case highlights an atypical and aggressive manifestation of HCC in a relatively younger, middle-aged man with only a brief two-year history of hepatitis B. The patient presented with a rapidly enlarging abdominal mass, upper gastrointestinal bleeding, and varicocele, features not commonly associated with the initial diagnosis of HCC.

Most HCCs are diagnosed through surveillance in high-risk populations and are often detected at smaller sizes [5]. This patient, however, presented with a massive exophytic tumor measuring nearly 19 cm, showing evidence of local invasion and intrahepatic metastases. The rapid progression and size at the time of presentation indicate a particularly aggressive tumor biology. Such advanced cases may be more frequently encountered in hepatitis B-endemic regions, where regular screening is often unavailable or delayed, leading to late diagnoses.

The tumor's growth pattern, arising exophytically from the left lobe of the liver, is particularly rare. HCC usually remains confined within the liver parenchyma and does not commonly present as a large, palpable epigastric mass [6]. In this case, the mass exerted a significant mass effect, compressing adjacent vascular structures, likely contributing to the development of a secondary varicocele due to impaired venous drainage. The elevated AFP level further supported the diagnosis, as extremely high AFP levels are associated with tumor burden and poor differentiation. Imaging features, including arterial enhancement and delayed venous washout on triphasic CT, were consistent with classic HCC and critical for diagnosis and treatment planning.

Additional findings, such as esophageal varices and hematemesis, indicated portal hypertension, which may have resulted from early fibrosis or compression of vascular structures by the tumor. While cirrhosis was not evident on imaging, these signs highlight the importance of gastrointestinal evaluation in hepatitis B patients with bleeding. Histopathology confirmed a poorly differentiated HCC, a subtype linked to higher metastatic potential and poorer therapeutic response [7]. Lymph node involvement and multifocal hepatic lesions confirmed the advanced stage. Given the limited curative options, a multidisciplinary approach was essential. Surgical debulking combined with systemic therapy was recommended to reduce tumor burden and enhance quality of life. This case reinforces the need for early HCC screening in hepatitis B-positive individuals and highlights the potential for aggressive disease even in the absence of classical risk factors or cirrhosis.

A key limitation of this case report is the unavailability of certain imaging modalities, including the initial ultrasound, scrotal Doppler, and EGD images. These were performed at the time of diagnosis but could not be retrieved due to retrospective case documentation limitations and restricted access to imaging archives at the referring facility. Despite this, the available triphasic CT scan and histopathological slide provide robust diagnostic support and illustrate the core findings. Future case documentation should prioritize comprehensive image retrieval to enhance educational and diagnostic clarity.

## Conclusions

This case underscores the importance of considering HCC in the differential diagnosis of rapidly growing abdominal masses, even in relatively young patients without cirrhosis or long-standing liver disease. The unusual presentation with an exophytic liver mass, complicated varicocele, and gastrointestinal bleeding reflects the aggressive nature of certain HCC variants. Early suspicion, comprehensive imaging, and multidisciplinary evaluation are critical for timely diagnosis and intervention. Clinicians should maintain a high index of suspicion for atypical HCC presentations in hepatitis B-positive individuals, as early recognition can significantly impact management and patient outcomes.

## References

[1] Chidambaranathan-Reghupaty S, Fisher PB, Sarkar D (2021). Hepatocellular carcinoma (HCC): epidemiology, etiology and molecular classification. Adv Cancer Res.

[2] Gana JC, Cifuentes LI, Gattini D, Villarroel Del Pino LA, Peña A, Torres-Robles R (2019). Band ligation versus beta-blockers for primary prophylaxis of oesophageal variceal bleeding in children with chronic liver disease or portal vein thrombosis. Cochrane Database Syst Rev.

[3] Balogh J, Victor D 3rd, Asham EH (2016). Hepatocellular carcinoma: a review. J Hepatocell Carcinoma.

[4] (2025). Hepatocellular carcinoma (HCC). https://emedicine.medscape.com/article/197319-overview?form=fpf.

[5] Bialecki ES, Di Bisceglie AM (2005). Diagnosis of hepatocellular carcinoma. HPB (Oxford).

[6] Sun VC, Sarna L (2008). Symptom management in hepatocellular carcinoma. Clin J Oncol Nurs.

[7] Rastogi A (2018). Changing role of histopathology in the diagnosis and management of hepatocellular carcinoma. World J Gastroenterol.

